# Perampanel outcomes at different stages of treatment in people with focal and generalized epilepsy treated in clinical practice: Evidence from the PERMIT study

**DOI:** 10.3389/fneur.2023.1120150

**Published:** 2023-03-30

**Authors:** Claudio Liguori, Estevo Santamarina, Adam Strzelczyk, Juan Jesús Rodríguez-Uranga, Rohit Shankar, Xiana Rodríguez-Osorio, Stéphane Auvin, Paolo Bonanni, Eugen Trinka, Rob McMurray, Ricardo Sáinz-Fuertes, Vicente Villanueva

**Affiliations:** ^1^Epilepsy Centre, Neurology Unit, University Hospital “Tor Vergata”, Rome, Italy; ^2^Department of Systems Medicine, University of Rome “Tor Vergata”, Rome, Italy; ^3^Epilepsy Unit, Neurology Department, Vall d'Hebron University Hospital, Barcelona, Spain; ^4^Epilepsy Center Frankfurt Rhine-Main, Department of Neurology, Goethe University, Frankfurt am Main, Germany; ^5^Centro de Neurología Avanzada, Sevilla, Spain; ^6^Peninsula School of Medicine, Plymouth, United Kingdom; ^7^Department of Neurology, Complexo Hospitalario Universitario de Santiago, Santiago, Spain; ^8^Université Paris Cité, INSERM NeuroDiderot, Paris, France; ^9^APHP, Robert Debré University Hospital, Pediatric Neurology Department, CRMR Epilepsies Rares, EpiCare Member, Paris, France; ^10^Institut Universitaire de France (IUF), Paris, France; ^11^Epilpesy and Clinical Neurophysiology Unit, Scientific Institute, IRCCS Eugenio Medea, Conegliano, Treviso, Italy; ^12^Department of Neurology, Christian-Doppler University Hospital, Paracelsus Medical University, Centre for Cognitive Neuroscience, Member of EpiCARE, Salzburg, Austria; ^13^Neuroscience Institute, Christian-Doppler University Hospital, Paracelsus Medical University, Centre for Cognitive Neuroscience, Salzburg, Austria; ^14^Institute of Public Health, Medical Decision-Making and HTA, UMIT–Private University for Health Sciences, Medical Informatics and Technology, Hall in Tyrol, Austria; ^15^Eisai Europe Ltd., Hatfield, United Kingdom; ^16^Refractory Epilepsy Unit, Hospital Universitario y Politécnico La Fe, Valencia, Spain

**Keywords:** clinical practice, early add-on therapy, effectiveness, focal epilepsy, generalized epilepsy, observational study, perampanel, tolerability

## Abstract

**Introduction:**

The PERMIT study is the largest pooled analysis of perampanel (PER) clinical practice data conducted to date.

**Methods:**

This *post-hoc* analysis of PERMIT investigated the effectiveness, safety and tolerability of PER when used as early add-on therapy (after failure of one or two previous antiseizure medications) in comparison with late add-on therapy (after failure of three or more previous antiseizure medications). Retention and effectiveness were assessed after 3, 6, and 12 months, and at the last visit (last observation carried forward). Effectiveness was assessed by seizure type (total seizures, focal seizures, generalized tonic-clonic seizures [GTCS]) and assessments included seizure freedom rate and responder rate. Safety and tolerability were assessed by evaluating adverse events (AEs) and discontinuation due to AEs.

**Results:**

The Full Analysis Set included 1184 and 2861 PWE treated with PER as early and late add-on therapy, respectively. Compared to the late add-on subgroup, the early add-on subgroup was characterized by later mean age at epilepsy onset, shorter mean duration of epilepsy, lower rates of intellectual disability and psychiatric comorbidity, and lower frequency of seizures per month, suggesting a less severe form of epilepsy in this subgroup. After 12 months, retention was significantly higher in the early versus late add-on subgroup (67.7% vs. 62.4%; *p* = 0.004). At the last visit, responder rates in the early versus late add-on subgroup were significantly higher for total seizures (68.2% vs. 39.3%; *p* < 0.001), focal seizures (65.0% vs. 36.8%; *p* < 0.001) and GTCS (83.7% vs. 67.2%; *p* < 0.001), as were seizure freedom rates (total seizures, 35.9% vs. 11.9% [*p* < 0.001]; focal seizures, 29.4% vs. 8.7% [*p* < 0.001]; GTCS, 69.0% vs. 48.1% [*p* < 0.001]). Incidence of AEs was significantly lower in the early versus late add-on subgroup (42.1% vs. 54.7%; *p* < 0.001), as was the rate of discontinuation due to AEs over 12 months (15.0% vs. 18.1%; *p* = 0.031).

**Discussion:**

This study demonstrated that PER was effective and generally well tolerated when initiated as early or late add-on therapy, but it was significantly more effective and better tolerated when initiated early. These findings support PER's use as a broad-spectrum, early add-on therapy for use in PWE with focal and generalized seizures.

## Introduction

In approximately 50% of people with epilepsy (PWE) initial monotherapy with an antiseizure medication (ASM) fails to result in seizure freedom (1). In such cases, management options are either to switch to an alternative ASM monotherapy, or to initiate add-on treatment with another ASM (2). Current evidence suggests that these two approaches result in similar efficacy and tolerability outcomes (3, 4). The likelihood of seizure freedom decreases with each additional add-on ASM (particularly for the first to third add-on ASMs) (1). This results in many PWE being treated with a large number of previous and concomitant ASMs before their seizures are considered adequately controlled. Although ~70% of PWE ultimately achieve seizure freedom, ~30% continue to have uncontrolled seizures (5), with a high burden of disease, increased morbidity and mortality (6, 7). Importantly, increased drug load is associated with an increased likelihood of adverse events (AEs) (8–10).

Perampanel (PER), a non-competitive α-amino-3-hydroxy-5-methyl-4-isoxazolepropionic acid (AMPA) receptor antagonist (11, 12), is approved in Europe for the adjunctive treatment of focal-onset seizures and generalized tonic-clonic seizures (GTCS) (13) and in the USA for the treatment of focal-onset seizures and the adjunctive treatment of GTCS (14). Approvals of PER were based on a comprehensive clinical trial program (15–19) which demonstrated PER's efficacy for both focal and generalized-onset seizures, suggesting its use as a broad-spectrum ASM (20, 21). These trials all included PWE who had previously failed treatment with at least two prior ASMs and/or were still experiencing seizures despite currently taking stable doses of one to three ASMs before initiation of PER (15–19). Clinical trial evidence for the use of PER as an early add-on treatment is therefore limited.

Real-world clinical practice data on PWE complement evidence from clinical trials by providing data on those who are more diverse in terms of clinical characteristics than those recruited for clinical trials, and provide additional information on the individualized treatment strategies employed in clinical practice (22–24). The PERaMpanel pooled analysIs in effecTiveness and tolerability (PERMIT) study is the largest pooled analysis of PER clinical practice data conducted to date, including data from ~5,200 PWE treated with PER for focal or generalized epilepsy (25). The large size of the PERMIT cohort allows meaningful subgroup analyses to be conducted. The objective of this study was to investigate the effectiveness, safety and tolerability of PER when used according to its Summary of Product Characteristics (13) as early add-on therapy in comparison with late add-on therapy, using data from the PERMIT study.

## Methods

### Study design

The PERMIT study was a pooled analysis of real-world data from 44 prospective, retrospective and cross-sectional studies and work groups in which PWE with focal and generalized epilepsy were treated with PER, full details of which have been published previously (25). Effectiveness was assessed after 3, 6, and 12 months of PER treatment and at final follow-up (i.e., the last observation of each individual, independent of when it occurred [last observation carried forward]; defined as “last visit”). Safety and tolerability were assessed for the duration of PER treatment. Each study included in PERMIT was approved by its own independent ethics committee and letters were sent to these ethics committees to inform them about the PERMIT study; as per current legislation, additional ethics committee approval was not required for participation in PERMIT (25). All PWE gave their informed consent prior to inclusion in the studies, according to the protocol.

A *post-hoc* subgroup analysis was conducted to compare outcomes in PWE who were treated at baseline with PER as early add-on therapy (defined as the addition of PER after the failure of one or two previous ASMs; “early add-on subgroup”) vs. PWE who were treated with PER as late add-on therapy (defined as initiation of PER after the failure of three or more previous ASMs; “late add-on subgroup”). Additional subanalyses were conducted to compare the effectiveness, safety and tolerability of PER when used as a first add-on therapy (defined as addition of PER after the failure of one previous ASM; “first add-on subgroup”) in comparison with a second add-on therapy (defined as initiation of PER after the failure of two previous ASMs; “second add-on subgroup”).

### Study population

The studies included in the PERMIT study analysis employed broad inclusion/exclusion criteria, to be representative of PWE encountered in clinical practice (25). The current analysis included all PWE for whom PER was initiated after the failure of one or more previous ASMs and who were treated with PER according to its Summary of Product Characteristics (13).

### Study assessments

Retention was assessed after 3, 6 and 12 months of PER treatment. Effectiveness was assessed by seizure type (total seizures, focal seizures, GTCS). Effectiveness assessments comprised seizure freedom rate, responder rate, and the proportion of PWE with worsening seizure frequency. Seizure freedom was defined as no seizures since at least the prior visit (either 3 or 6 months, depending on the timepoint at which seizure freedom was assessed), and response was defined as ≥50% seizure frequency reduction from baseline (i.e., prior to PER initiation). Since the definition of “baseline” differed between studies included in PERMIT, baseline seizure frequency was standardized as number of seizures per month. Safety and tolerability were assessed by evaluating AEs, AEs leading to discontinuation, psychiatric AEs, and psychiatric AEs in PWE who discontinued. Information relating to PER dosing and use of concomitant ASMs was also assessed. All assessments were compared for the early vs. late add-on subgroups.

### Additional subgroup analysis

Additional analyses were conducted to compare retention, effectiveness (responder rate and seizure freedom rate for total seizures only), and safety/tolerability in the first vs. second add-on subgroups.

### Statistical analyses

Details of the statistical methodology employed in PERMIT has been published previously (25). The Full Analysis Set (FAS) included all PWE treated with PER. The Retention Population included PWE from the FAS whose PER status was known at some point during the first 12 months after starting treatment (including those with ongoing PER treatment at 12 months, those who stopped PER prior to 12 months and those lost to follow-up/end of study follow-up prior to 12 months). The Effectiveness Population included PWE from the FAS who had at least one effectiveness measurement available. The Tolerability Population included PWE from the FAS for whom data on AEs were available.

There was great heterogeneity in the objectives of each study included in the pooled analysis and therefore in the information reported. As previously described, PERMIT attempted to combine reported information in the most complete way possible (25). Missing data were not imputed, except in cross-sectional studies, in which the last visit datum was captured to include in the established cut-off points (3, 6 or 12 months). When an observation timepoint did not match the established cut-off points, the following allocations were made: observations performed between 1.5 and 4.5 months were allocated to the 3-month visit; those performed between 4.5 and 9 months were allocated to the 6-month visit; and those performed between 9 and 15 months were allocated to the 12-month visit. A “final” variable was created in which the last observation of each PWE was included, independently of when it occurred (defined as “last visit”). No hypothesis was defined, and no systematic review of individual PWE was considered, due to the heterogeneity of individual samples and the different objectives of each study; therefore, individual studies were not treated as clusters.

Quantitative variables were described as mean, standard deviation (SD), median, minimum and maximum values, together with the number of valid cases and confidence intervals (CIs) or interquartile range (25^th^ percentile to 75^th^ percentile). Qualitative variables were described as absolute frequencies and percentages. Data were not available for all PWE at every timepoint; therefore, for each variable, the total number of PWE for whom the datum in question was available was stated and used as the denominator for frequency analyses. Retention (on PER treatment) was studied within the first 12 months of follow-up using Kaplan–Meier methodology. Time to PER discontinuation was compared between the early vs. late add-on subgroups, and first vs. second add-on subgroups, using the Log Rank test. Between-group comparisons of baseline characteristics, retention, effectiveness outcomes and safety/tolerability assessments for the early vs. late add-on subgroups were conducted using the Chi-squared test, Student's *t*-test, Mann-Whitney U test, or Fisher's exact test, as appropriate. Between-group comparisons of retention, effectiveness outcomes and safety/tolerability assessments for the first vs. second add-on subgroups were conducted using the Chi-squared test. The significance level was set at 5% and the statistical package SPSS 25.0 was used for all analyses.

## Results

The PERMIT study collected information from 5,200 PWE with epilepsy who had initiated PER treatment and the final FAS included 5,193 PWE (25). The current study included 4,045 PWE from the PERMIT FAS who were treated according to PER's Summary of Product Characteristics (13) as add-on therapy having been treated with at least one previous ASM. Of these 4,045 PWE, 1,184 initiated PER as early add-on therapy and 2,861 initiated PER as late add-on therapy. The Retention Population included 3,885 PWE (early add-on, *n* = 1,137; late add-on, *n* = 2,748), the Effectiveness Population included 3,928 PWE (early add-on, *n* = 1,140; late add-on, *n* = 2,788) and the Tolerability Population included 3,666 PWE (early add-on, *n* = 1,103; late add-on, *n* = 2,563).

### Study population

In the overall study population, 50.1% of PWE were female, the mean (SD) age was 40 (15.2) years, and the mean (SD) epilepsy duration was 24.1 (16.1) years (Table 1). Seizure types at baseline were focal seizures only (77.2%), GTCS only (22.7%), and both focal seizures and GTCS (0.1%). There were significant differences in the demographic and baseline characteristics of the early vs. late add-on subgroups (Table 1). For the early vs. late add-on subgroups, the proportion of male PWE was significantly higher (53.7 vs. 48.3%; *p* = 0.002), mean age at epilepsy onset was significantly later (25.9 vs. 12.3 years; *p* < 0.001), and the mean duration of epilepsy was significantly shorter (14.2 vs. 27.6 years; *p* < 0.001). Etiology was significantly different between subgroups (*p* < 0.001), most notably with a higher proportion of PWE with a genetic etiology in the early vs. late add-on subgroup (17.7 vs. 9.3%). The proportion of PWE with intellectual disability was significantly lower in the early vs. late add-on subgroup (13.7 vs. 23.4%; *p* < 0.001), as was the proportion of PWE with psychiatric comorbidity (19.0 vs. 26.1%; *p* < 0.001). Baseline monthly seizure frequency was significantly lower in the early vs. late add-on subgroup for total seizures (mean, 6.9 vs. 11.9; *p* = 0.029) and focal seizures (mean, 9.1 vs. 18.0; *p* < 0.001). As expected, the mean number of previous ASMs was significantly lower in the early vs. late add-on subgroup (1.5 vs. 6.7; *p* < 0.001), as was the number of concomitant ASMs at baseline (1.4 vs. 2.7; *p* < 0.001).

**Table 1:** Demographic and baseline characteristics (full analysis set).

**Characteristic**	**Total *N* = 4,045**	**Early add-on *N* = 1,184**	**Late add-on *N* = 2,861**	***p*-value^a^**
**Gender**				
N^b^ Female, *n* (%) Male, *n* (%)	4,036 2,022 (50.1) 2,014 (49.9)	1,181 547 (46.3) 634 (53.7)	2,855 1,475 (51.7) 1,380 (48.3)	0.002^c^
**Age**				
N^b^ Mean (SD), years Median (range), years	4,045 40.0 (15.2) 39.0 (4.0–97.0)	1,184 40.2 (17.2) 38.0 (4.0–97.0)	2,861 39.9 (14.3) 39.0 (5.0–86.0)	NS^d^
**Age category**				
N^b^ 4– < 12 years, *n* (%) ≥12– < 18 years, *n* (%) ≥18–64 years, *n* (%) ≥65 years, *n* (%)	4,045 17 (0.4) 179 (4.4) 3,580 (88.5) 269 (6.7)	1,184 5 (0.4) 69 (5.8) 989 (83.5) 121 (10.2)	2,861 12 (0.4) 110 (3.8) 2,591 (90.6) 148 (5.2)	–
**Age at epilepsy onset**				
N^b^ Mean (SD), years Median (range), years	3,504 15.9 (16.5) 12.0 (0.0–97.0)	917 25.9 (20.2) 20.0 (0.0–97.0)	2,587 12.3 (13.2) 9.2 (0.0–81.0)	< 0.001^e^
**Duration of epilepsy**				
N^b^ Mean (SD), years Median (range), years	3,504 24.1 (16.1) 22.0 (0.0–82.0)	917 14.2 (13.7) 10.0 (0.0–68.0)	2,587 27.6 (15.5) 26.0 (0.0–82.0)	< 0.001^e^
**Etiology** ^f^				
N^b^ Structural, *n* (%) Genetic, *n* (%) Infectious, *n* (%) Immune, *n* (%) Unknown, *n* (%) Other, *n* (%)	3,072 1,636 (53.3) 343 (11.2) 88 (2.9) 24 (0.8) 980 (31.9) 1 (< 0.1)	690 351 (50.9) 122 (17.7) 11 (1.6) 4 (0.6) 201 (29.1) 1 (0.1)	2,382 1,285 (53.9) 221 (9.3) 77 (3.2) 20 (0.8) 779 (32.7) 0	< 0.001^c^
**Intellectual disability**				
N^b^ Yes, *n* (%) No, *n* (%)	2,007 435 (21.7) 1,572 (78.3)	365 50 (13.7) 315 (86.3)	1,642 385 (23.4) 1,257 (76.6)	< 0.001^c^
**Psychiatric comorbidity**				
N^b^ Yes, *n* (%) No, *n* (%)	2,041 478 (23.4) 1,563 (76.6)	759 144 (19.0) 615 (81.0)	1,282 334 (26.1) 948 (73.9)	< 0.001^c^
**Type of psychiatric comorbidity**				
N^b^ Depression, *n* (%) Anxiety, *n* (%) Psychosis, *n* (%) Hyperactivity, *n* (%) Autism, *n* (%) Personality disorder, *n* (%) Irritability, *n* (%) Behavioral disorder, *n* (%) Others, *n* (%)	2,041 90 (4.4) 73 (3.6) 23 (1.1) 18 (0.9) 15 (0.7) 11 (0.5) 7 (0.3) 6 (0.3) 22 (1.1)	759 40 (5.3) 33 (4.3) 8 (1.1) 9 (1.2) 10 (1.3) 5 (0.7) 2 (0.3) 0 7 (0.9)	1,282 50 (3.9) 40 (3.1) 15 (1.2) 9 (0.7) 5 (0.4) 6 (0.5) 5 (0.4) 6 (0.5) 15 (1.2)	–
**Seizure type**				
N^b^ Focal seizures only, *n* (%) GTCS only, *n* (%) Both focal seizures and GTCS, *n* (%)	1,414 1,091 (77.2) 321 (22.7) 2 (0.1)	741 586 (79.1) 155 (20.9) 0 (0.0)	673 505 (75.0) 166 (24.7) 2 (0.3)	NS^c^ NS^c^ NS^g^
**Seizure frequency/month**				
***Total seizures***				
N^b^ Mean (SD) Median (range)	1,397 9.3 (29.1) 1.7 (0.0–600.0)	776 6.9 (20.6) 1.3 (0.0–300.0)	621 11.9 (35.9) 2.0 (0.0–600.0)	0.029^e^
***Focal seizures***				
N^b^ Mean (SD) Median (range)	980 13.0 (34.2) 4.0 (0.1–600.0)	549 9.1 (23.6) 2.3 (0.2–300.0)	431 18.0 (43.6) 5.3 (0.1–600.0)	< 0.001^e^
***GTCS***				
N^b^ Mean (SD) Median (range)	197 1.3 (2.7) 0.7 (0.0–300.0)	120 1.0 (1.5) 0.7 (0.0–12.0)	77 1.5 (3.4) 0.7 (0.0–30.0)	NS^e^
**Number of previous ASMs**				
N^b^ Mean (SD) Median (range)	3,255 5.7 (3.5) 5.0 (1–19)	646 1.5 (0.5) 2.0 (1–2)	2,609 6.7 (3.1) 6.0 (3–19)	< 0.001^e^
**Number of previous ASMs**				
N^b^ 1, *n* (%) 2, *n* (%) 3, *n* (%) 4, *n* (%) 5, *n* (%) 6, *n* (%) 7, *n* (%) 8, *n* (%) 9, *n* (%) ≥10, *n* (%)	3,255 295 (9.1) 351 (10.8) 359 (11.0) 386 (11.9) 360 (11.1) 364 (11.2) 258 (7.9) 229 (7.0) 189 (5.8) 464 (14.3)	646 295 (45.7) 351 (54.3) – – – – – – – –	2,609 – – 359 (13.8) 386 (14.8) 360 (13.8) 364 (14.0) 258 (9.9) 229 (8.8) 189 (7.2) 464 (17.8)	–
**Most frequently used**^h^ **previous ASMs**				
N^b^ Levetiracetam, *n* (%) Valproate, *n* (%) Carbamazepine, *n* (%) Lamotrigine, *n* (%) Topiramate, *n* (%) Clobazam, *n* (%) Oxcarbazepine, *n* (%) Zonisamide, *n* (%) Clonazepam, *n* (%) Lacosamide, *n* (%) Phenytoin, *n* (%)	1,086 656 (60.4) 531 (48.9) 440 (40.5) 369 (34.0) 307 (28.3) 238 (21.9) 225 (20.7) 223 (20.5) 212 (19.5) 203 (18.7) 194 (17.9)	419 199 (47.5) 99 (23.6) 62 (14.8) 69 (16.5) 16 (3.8) 8 (1.9) 29 (6.9) 22 (5.3) 7 (1.7) 30 (7.2) 16 (3.8)	667 457 (68.5) 432 (64.8) 378 (56.7) 300 (45.0) 291 (43.6) 230 (34.5) 196 (29.4) 201 (30.1) 205 (30.7) 173 (25.9) 178 (26.7)	–
**Number of concomitant ASMs**				
N^b^ Mean (SD) Median (range)	4,045 2.3 (1.0) 2.0 (1–7)	1,184 1.4 (0.5) 1.0 (1–2)	2,861 2.7 (1.0) 3.0 (1–7)	< 0.001^e^
**Number of concomitant ASMs**				
N^b^ 1, *n* (%) 2, *n* (%) 3, *n* (%) 4, *n* (%) 5, *n* (%) 6, *n* (%) 7, *n* (%)	4,045 924 (22.8) 1,507 (37.3) 1,134 (28.0) 392 (9.7) 68 (1.7) 16 (0.4) 4 (0.1)	1,184 652 (55.0) 532 (45.0) – – – – –	2,861 272 (9.5) 975 (34.1) 1,134 (39.6) 392 (13.7) 68 (2.4) 16 (0.6) 4 (0.1)	–
**Most frequently used**^h^ **concomitant ASMs**				
N^b^ Levetiracetam, *n* (%) Lamotrigine, *n* (%) Valproate, *n* (%) Carbamazepine, *n* (%) Lacosamide, *n* (%) Clobazam, *n* (%)	3,918 1,559 (39.8) 987 (25.2) 958 (24.5) 942 (24.0) 770 (19.7) 636 (16.2)	1,126 431 (38.3) 160 (14.2) 252 (22.4) 198 (17.6) 126 (11.2) 36 (3.2)	2,792 1,128 (40.4) 827 (29.6) 706 (25.3) 744 (26.6) 644 (23.1) 600 (21.5)	–

^a^For comparisons between the early vs. late add-on groups; ^b^Number of PWE for whom datum in question was available; ^c^Chi-squared test; ^d^Student's t-test; ^e^Mann-Whitney U test; ^f^International League Against Epilepsy 2017 classification; ^g^Fisher's exact test; ^h^≥20% of PWE in any group.

ASM, antiseizure medication; GTCS, generalized tonic-clonic seizures; NS, not significant; PWE, people with epilepsy; SD, standard deviation.

### Treatment

The PER dose at baseline was significantly higher in the early add-on subgroup (mean, 2.9 mg/day; SD, 1.5; median, 2.0; range, 1–10; *n* = 473) than in the late add-on subgroup (mean, 2.1 mg/day; SD, 0.5; median, 2.0; range, 1–8; *n* = 1,336) (*p* < 0.001). However, the PER dose at the last visit was significantly lower in the early add-on subgroup (mean, 5.8 mg/day; SD, 2.3; median, 6.0; range, 2–12; *n* = 960) than in the late add-on subgroup (mean, 6.7 mg/day; SD, 2.7; median, 6.0, range, 1–12; *n* = 1,757) (*p* < 0.001). In the early add-on group, PWE had been treated with one (45.7%) or two (54.3%) previous ASMs; most commonly, levetiracetam (47.5%) and valproate (23.6%) (Table 1). In the late add-on subgroup, PWE had been treated with 3–19 previous ASMs (mean, 6.7; median 6.0); most commonly, levetiracetam (68.5%), valproate (64.8%), carbamazepine (56.7%), lamotrigine (45.0%) and topiramate (43.6%). In the early add-on subgroup, the mean (SD) number of concomitant ASMs decreased from 1.4 (0.5) at baseline to 1.2 (0.5) at the last visit. The most used ASMs at baseline were levetiracetam (38.3%), valproate (22.4%) and carbamazepine (17.6%) (Table 1). In the late add-on subgroup, the mean (SD) number of concomitant ASMs decreased from 2.7 (1.0) at baseline to 2.4 (0.9) at the last visit. The most used ASMs at baseline were levetiracetam (40.4%), lamotrigine (29.6%) and carbamazepine (26.6%). In the overall study population, the mean (SD) number of concomitant ASMs decreased from 2.3 (1.0) at baseline to 2.0 (1.0) at the last visit.

### Retention (retention population)

**Figure 1:**
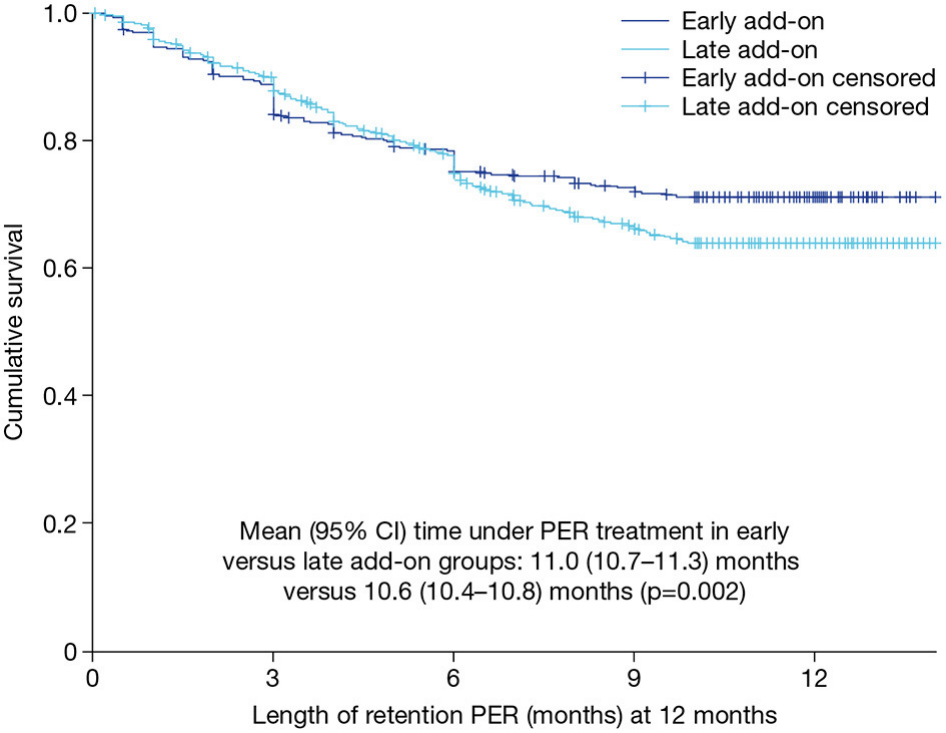
Kaplan–Meier curve for retention on PER treatment over 12 months in the early and late add-on subgroups (retention population). CI, confidence interval; PER, perampanel.

Retention rates in the early vs. late add-on subgroups were 89.5% (1,018/1,137) vs. 90.8% (2,496/2,748) at Month 3 (*p* = not significant), 78.2% (812/1,039) vs. 80.4% (2,140/2,663) at Month 6 (*p* = not significant), and 67.7% (648/957) vs. 62.4% (1,576/2,525) at Month 12 (*p* = 0.004). Over 12 months of follow-up, the mean (95% CI) time under PER treatment was 11.0 (10.7–11.3) months in the early add-on subgroup vs. 10.6 (10.4–10.8) months in the late add-on subgroup (*p* = 0.002) (Figure 1). In the early add-on subgroup, reasons for discontinuation of PER treatment at 12 months were intolerability (*n* = 130; 13.6%), lack of efficacy (*n* = 51; 5.3%), both intolerability and lack of efficacy (*n* = 14; 1.5%), seizure worsening (*n* = 2; 0.2%) and other reasons [*n* = 18; 1.9% (financial problems, *n* = 6; exitus, *n* = 5; pregnancy, *n* = 3; transferred to another hospital, *n* = 2; surgery, *n* = 1; unable to take medication–pneumonia, n=1)]; reasons for discontinuation were unknown for 94 PWE (9.8%). In the late add-on subgroup, reasons for discontinuation of PER treatment at 12 months were intolerability (*n* = 379; 15.0%), lack of efficacy (*n* = 278; 11.0%), both intolerability and lack of efficacy (*n* = 79; 3.1%), seizure worsening (*n* = 36; 1.4%) and other reasons [*n* = 7; 0.3% (exitus, *n* = 3; patient decision, *n* = 2; pregnancy, *n* = 1; poor compliance, *n* = 1)]; reasons for discontinuation were unknown for 170 PWE (6.7%).

### Effectiveness (effectiveness population)

In the overall study population, 98.3% (1,581/1,608) of PWE had seizures in the 3 months prior to initiating PER. There was no significant difference in the proportions of PWE with seizures in the early vs. late add-on subgroups [98.3% (820/834) vs. 98.3% (761/774)]. In the overall study population, 99.0% (1,080/1,091) of PWE with a history of focal seizures experienced focal seizures in the 3 months prior to initiating PER, and there was no significant difference in the proportions of PWE with focal seizures in the early vs. late add-on subgroups [99.1% (581/586) vs. 98.8% (499/505)]. In the overall study population, 95.0% (305/321) of PWE with a history of GTCS experienced GTCS in the 3 months prior to initiating PER, and, again, there was no significant difference in the proportions of PWE with GTCS in the early vs. late add-on subgroups [94.2% (146/155) vs. 95.8% (159/166)].

**Figure 2:**
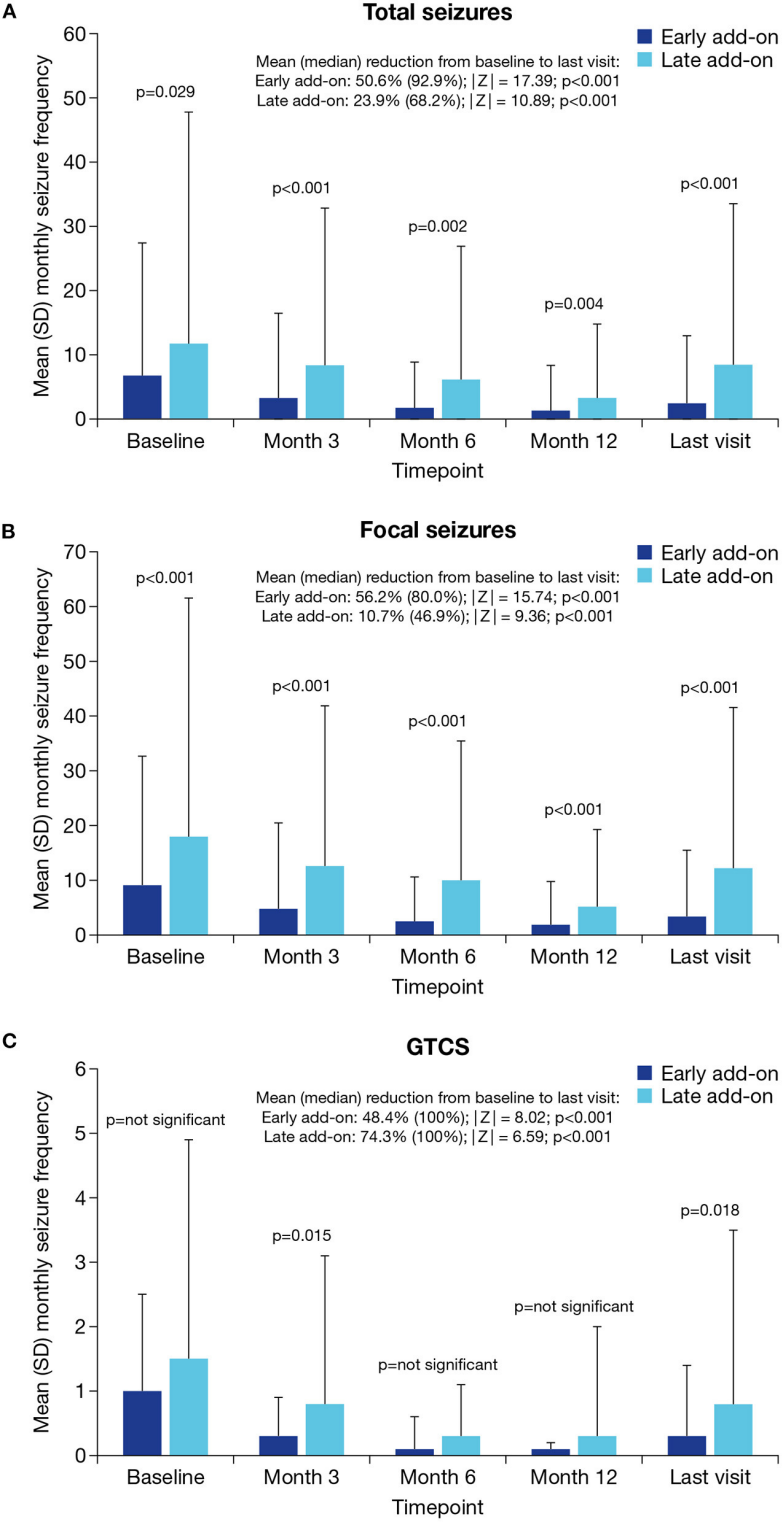
Mean monthly seizure frequency at baseline, Month 3, Month 6, Month 12 and the last visit in the early and late add-on subgroups for **(A)** total seizures, **(B)** focal seizures and **(C)** GTCS (effectiveness population). GTCS, generalized tonic-clonic seizures; SD, standard deviation.

The monthly frequency of total seizures, focal seizures and GTCS decreased significantly from baseline to last visit in both the early and late add-on subgroups (*p* < 0.001 for all), but the decrease was greater in the early vs. late add-on subgroup for total seizures (median decrease, 92.9 vs. 68.2%) and focal seizures (median decrease, 80.0 vs. 46.9%); for GTCS, the median decrease was 100% in both subgroups (Figure 2). The monthly frequency of total seizures was significantly lower in the early vs. late add-on subgroup at baseline and all subsequent timepoints (*p* < 0.05 for all) (Figure 2A). The monthly frequency of focal seizures was also significantly lower in the early vs. late add-on subgroup at baseline and all subsequent timepoints (*p* < 0.001 for all) (Figure 2B). The monthly frequency of GTCS was significantly lower in the early vs. late add-on subgroup at Month 3 (*p* = 0.015) and the last visit (*p* = 0.018), but the frequency did not differ significantly between the subgroups at baseline, Month 6 or Month 12 (Figure 2C).

**Figure 3:**
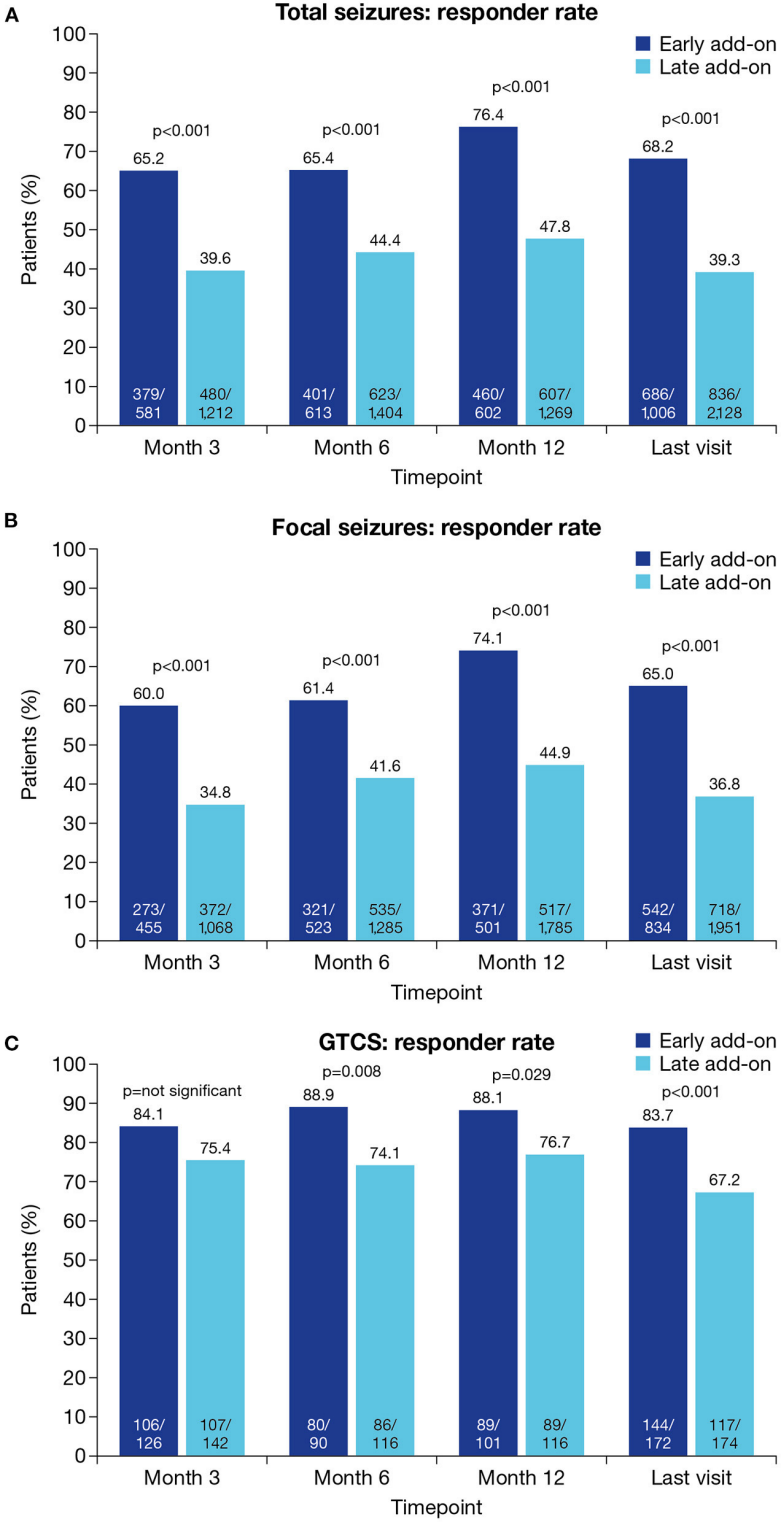
Responder rates at Month 3, Month 6, Month 12 and the last visit in the early and late add-on subgroups for **(A**) total seizures, **(B**) focal seizures and **(C)** GTCS (effectiveness population). Response was defined as ≥50% seizure frequency reduction from baseline. GTCS, generalized tonic-clonic seizures.

Responder rates for total seizures were significantly higher in the early vs. late add-on subgroup at all timepoints (*p* < 0.001 for all; Figure 3A). At the last visit, total seizure responder rates in the early vs. late add-on subgroups were 68.2% (686/1,006) vs. 39.3% (836/2,128) (*p* < 0.001). Similarly, responder rates for focal seizures were significantly higher in the early vs. late add-on subgroup at all timepoints (*p* < 0.001 for all; Figure 3B). At the last visit, focal seizure responder rates in the early vs. late add-on subgroups were 65.0% (542/834) vs. 36.8% (718/1,951) (*p* < 0.001). Responder rates for GTCS were significantly higher in the early vs. late add-on subgroup at Month 6 (*p* = 0.008), Month 12 (*p* = 0.029) and the last visit (*p* < 0.001) but did not differ significantly between subgroups at Month 3 (Figure 3C). At the last visit, responder rates for GTCS in the early vs. late add-on subgroups were 83.7% (144/172) vs. 67.2% (117/174) (*p* < 0.001).

**Figure 4:**
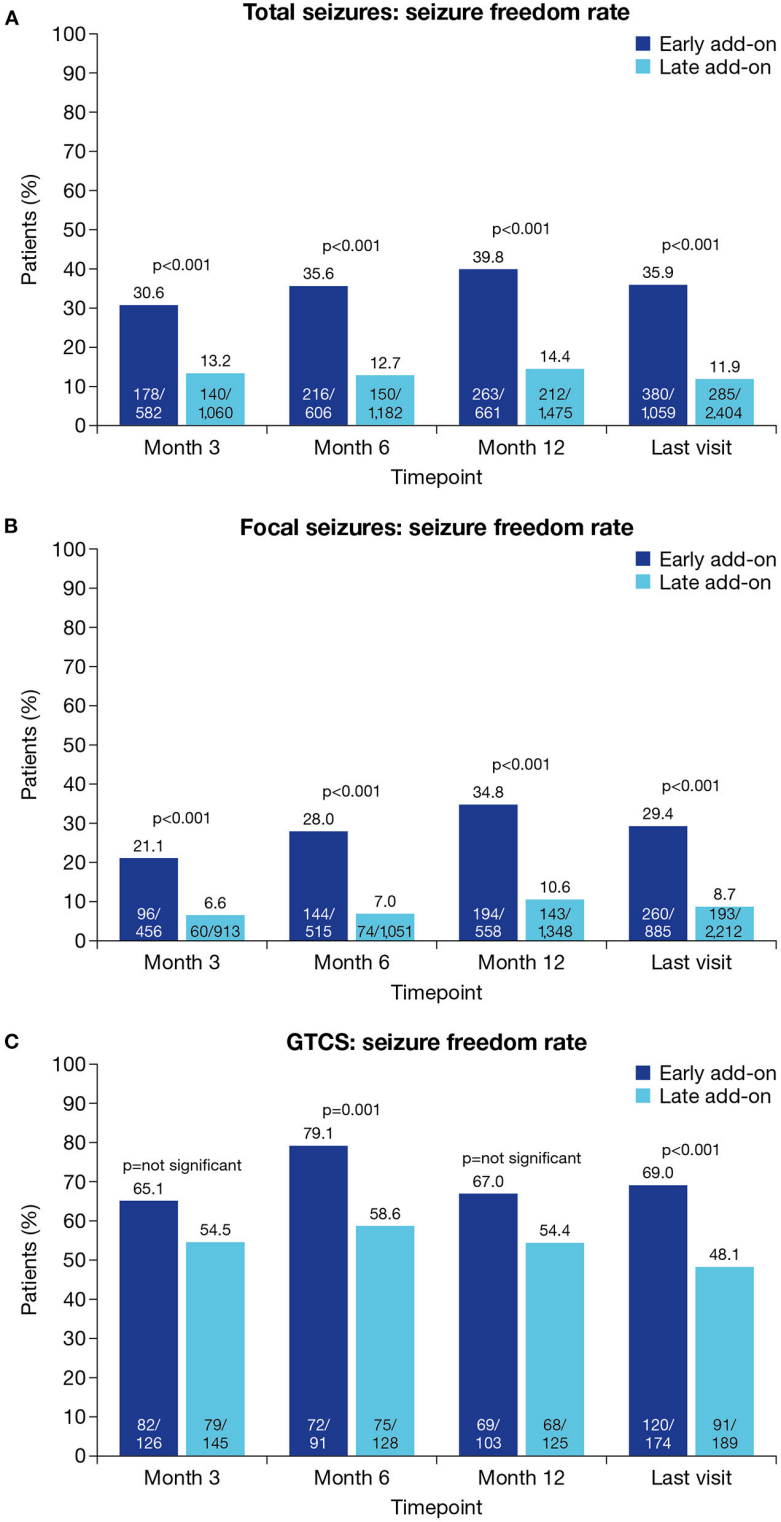
Seizure freedom rates at Month 3, Month 6, Month 12 and the last visit in the early and late add-on subgroups for **(A)** total seizures, **(B)** focal seizures and **(C)** GTCS (effectiveness population). Seizure freedom was defined as no seizures since at least the prior visit. GTCS, generalized tonic-clonic seizures.

Seizure freedom rates for total seizures were significantly higher in the early vs. late add-on subgroup at all timepoints (*p* < 0.001 for all; Figure 4A). At the last visit, seizure freedom rates for total seizures in the early vs. late add-on subgroups were 35.9% (380/1,059) vs. 11.9% (285/2,404) (*p* < 0.001). Similarly, seizure freedom rates for focal seizures were significantly higher in the early vs. late add-on subgroup at all timepoints (*p* < 0.001 for all; Figure 4B). At the last visit, seizure freedom rates for focal seizures in the early vs. late add-on subgroups were 29.4% (260/885) vs. 8.7% (193/2,212) (*p* < 0.001). Seizure freedom rates for GTCS were significantly higher in the early vs. late add-on subgroup at Month 6 (*p* = 0.001) and the last visit (*p* < 0.001) but did not differ between subgroups at Month 3 or Month 12 (Figure 4C). At the last visit, seizure freedom rates for GTCS in the early vs. late add-on subgroups were 69.0% (120/174) vs. 48.1% (91/189) (*p* < 0.001).

The proportions of PWE with worsening total seizure frequency were significantly lower in the early vs. late add-on subgroup at Month 3, Month 12 and the last visit (*p* ≤ 0.001 for all) but did not differ significantly between subgroups at Month 6 (Supplementary Figure 1A). At the last visit, the proportions of PWE with worsening total seizure frequency in the early vs. late add-on subgroups were 6.2% (61/979) vs. 13.0% (274/2,105) (*p* < 0.001). Similarly, the proportions of PWE with worsening focal seizure frequency were significantly lower in the early vs. late add-on subgroup at Month 3, Month 12 and the last visit (*p* ≤ 0.001 for all) but did not differ significantly between subgroups at Month 6 (Supplementary Figure 1B). At the last visit, the proportions of PWE with worsening focal seizure frequency in the early vs. late add-on subgroups were 6.4% (52/808) vs. 13.7% (264/1,928) (*p* < 0.001). The proportions of PWE with worsening GTCS frequency did not differ significantly between subgroups at any timepoint (Supplementary Figure 1C). At the last visit, the proportions of PWE with worsening GTCS frequency in the early vs. late add-on subgroups were 5.3% (9/171) vs. 5.7% (10/174).

### Safety and tolerability (tolerability population)

The proportion of PWE with AEs was significantly lower for PWE in the early vs. late add-on subgroup [42.1% (464/1,103) vs. 54.7% (1,401/2,563); *p* < 0.001] (Table 2). The most frequently reported AEs in the early vs. late add-on subgroups (≥5% of PWE in either subgroup) were dizziness/vertigo (9.4 vs. 13.7%), somnolence (7.3 vs. 9.3%) and irritability (8.3 vs. 5.5%). Over 12 months, AEs led to discontinuation of a significantly lower proportion of PWE in the early versus late add-on subgroup [15.0% (144/957) vs. 18.1% (458/2,525); *p* = 0.031], and the most frequently reported AEs leading to discontinuation (≥1% of PWE in either subgroup) were dizziness/vertigo (3.1 vs. 4.2%), somnolence (2.0 vs. 2.7%), irritability (3.0 vs. 2.1%) and behavioral disorders (0.6 vs. 2.1%). The proportion of PWE with psychiatric AEs was similar for the early vs. late add-on subgroups [19.4% (213/1,099) vs. 21.0% (534/2,543); *p* =not significant], as was the proportion of PWE with psychiatric AEs who discontinued [7.7% (82/1,066) vs. 9.4% (235/2,491); *p* = not significant]. The most frequently reported psychiatric AE (≥5% of PWE in either subgroup) was irritability, which was reported for 8.3 and 5.5% of PWE in the early and late add-on subgroups, respectively. The most frequently reported psychiatric AEs in PWE who discontinued (≥1% of PWE in either subgroup) in the early vs. late add-on subgroups were irritability (3.0 vs. 2.1%) and behavioral disorders (0.6 vs. 2.1%).

**Table 2:** Summary of safety and tolerability (tolerability population).

	**Early add-on *N* = 1,103**	**Late add-on *N* = 2,563**	***p*-value^a^**
PWE with any AE
n (%)	464 (42.1)	1401 (54.7)	< 0.001^b^
**Most frequently reported AEs**,^c^ **n (%)**			
Dizziness/vertigo Somnolence Irritability Behavioral disorders Instability/ataxia Fatigue Apathy	104 (9.4) 80 (7.3) 91 (8.3) 8 (0.7) 18 (1.6) 17 (1.5) 22 (2.0)	350 (13.7) 239 (9.3) 141 (5.5) 113 (4.4) 68 (2.7) 57 (2.2) 11 (0.4)	–
**PWE with AEs leading to discontinuation**			
n (%)	144 (15.0)^d^	458 (18.1)^e^	0.031^b^
**Most frequently reported AEs leading to discontinuation**,^f^ **n (%)**			
Dizziness/vertigo Somnolence Irritability Behavioral disorders	30 (3.1)^d^ 19 (2.0)^d^ 29 (3.0)^d^ 6 (0.6)^d^	107 (4.2)^e^ 67 (2.7)^e^ 52 (2.1)^e^ 52 (2.1)^e^	–
**PWE with any psychiatric AE**			
n (%)	213 (19.4)^g^	534 (21.0)^h^	NS^b^
**Most frequently reported psychiatric AEs**,^f^ **n (%)**			
Irritability Behavioral disorders Mood disturbance Aggression Anxiety Apathy	91 (8.3)^g^ 8 (0.7)^g^ 4 (0.4)^g^ 13 (1.2)^g^ 17 (1.5)^g^ 22 (2.0)^g^	141 (5.5)^h^ 113 (4.4)^h^ 37 (1.4)^h^ 24 (0.9)^h^ 20 (0.8)^h^ 11 (0.4)^h^	–
**PWE with psychiatric AEs who discontinued** ^i^			
n (%)	82 (7.7)^j^	235 (9.4)^k^	NS^b^
**Most frequently reported psychiatric AEs**^f^ **in PWE who**			
**discontinued**,^i^ **n (%)**			
Irritability Behavioral disorders	29 (2.7)^j^ 6 (0.6)^j^	52 (2.1)^k^ 52 (2.1)^k^	–

^a^For comparisons between the early vs. late add-on groups; ^b^Chi-squared test; ^c^≥2% of PWE in either group; ^d^N = 957; ^e^N = 2,525; ^f^≥1% of PWE in either group; ^g^N = 1,099; ^h^N = 2,543; ^i^These PWE had psychiatric AEs but it was not possible to determine if it was these AEs that led to discontinuation; ^j^N = 1,066; ^k^N = 2,491.

AE, adverse event; NS, not significant; PWE, people with epilepsy.

### Additional subgroup analysis

Of the 1,184 PWE in the early add-on subgroup (FAS), the number of previous ASMs was known for 646 PWE, of whom 295 (45.7%) initiated PER as first add-on therapy and 351 (54.3%) initiated PER as second add-on therapy. Retention rates did not differ significantly for the first vs. second add-on subgroups at any timepoint (Supplementary Figure 2A). Responder rates for total seizures were significantly higher in the first vs. second add-on subgroup only at Month 12 (*p* = 0.006) and the last visit (*p* = 0.026; Supplementary Figure 2B). Seizure freedom rates for total seizures did not differ between subgroups (Supplementary Figure 2C). The proportion of PWE with AEs did not differ significantly between the first vs. second add-on subgroup [43.2% (121/280) vs. 44.8% (145/324)]. Similarly, the proportion of PWE who discontinued due to AEs over 12 months did not differ significantly between the first vs. second add-on subgroup [15.5% (41/264) vs. 14.3% (41/286)].

## Discussion

This *post-hoc* analysis of the PERMIT study was conducted to compare the effectiveness and safety/tolerability of PER when used in everyday clinical practice as early vs. late add-on therapy in PWE with focal and generalized seizures. PER was effective in both treatment settings, with significant reductions from baseline to last visit in the monthly frequencies of total seizures, focal seizures and GTCS in both subgroups. However, PER was significantly more effective in the early vs. late add-on subgroup, in terms of monthly seizure frequency reduction, responder rate and seizure freedom rate, across seizure types. Rates of total and focal seizure worsening were also significantly lower in the early vs. late add-on subgroup at most timepoints, while rates of GTCS worsening were low in both subgroups. Further analysis of the early add-on subgroup demonstrated that PER was effective when used as first or second add-on therapy. Although retention and seizure freedom rates for total seizures did not differ significantly between the first and second add-on subgroups, responder rates for total seizures were significantly higher in the first vs. second add-on subgroup at Month 12 and the last visit, indicating that PER may be particularly effective in the first add-on setting. An Italian consensus clinical practice statement has already acknowledged that PER has many features that justify its use as a first add-on therapy (26), and, taken together, the findings of the current study provide evidence to support these recommendations. PER was particularly effective in PWE with GTCS, regardless of whether it was used as early or late add-on therapy, supporting its use as a broad-spectrum ASM (21, 27).

PER was generally well-tolerated when used in the early and late add-on settings and the AEs reported were consistent with its known safety profile (13). However, PER was significantly better tolerated in the early vs. late add-on subgroup, in terms of incidence of AEs and rate of discontinuation due to AEs. Further analysis of the early add-on subgroup found no significant differences in the incidence of AEs and rate of discontinuation due to AEs when PER was used as first and second add-on therapy. The proportion of PWE with psychiatric AEs and the proportion of PWE with psychiatric AEs who discontinued did not differ significantly between the early and late add-on subgroups, although the frequencies of both endpoints were numerically lower in the former vs. latter subgroup, perhaps indicating a particular or idiosyncratic sensitivity to these AEs in the late add-on subgroup, regardless of other factors. Psychiatric AEs have been reported as a common side effect in PER clinical trials (defined as affecting ≥1/100 to < 1/10 PWE) (13). The higher incidence of psychiatric AEs reported in the current study is likely due to the fact that approximately a quarter of PWE (23.4%) had psychiatric comorbidities at baseline, since bivariable analysis of the overall PERMIT population previously found a significant association between the incidence of psychiatric AEs and the presence of previous psychiatric comorbidity (25). By contrast, PWE with psychiatric comorbidities are typically excluded from participating in clinical trials (22, 24).

Retention rates were high in both subgroups, but 12-month retention was significantly higher in the early vs. late add-on subgroup, indicating more favorable overall effectiveness and tolerability in the early subgroup. The rate of discontinuation due to lack of efficacy was approximately twice as high in the late vs. early add-on subgroup, consistent with a higher rate of treatment refractoriness in the late add-on subgroup. Retention rates at 12 months in both the first (73.1%) and the second add-on (72.0%) were higher than those reported in previous studies of PER. In a Pan-European pooled analysis study of 2,396 PWE the retention rate at 12 months was 48% (28) and in a 2-year observational study of 122 PWE conducted in Austria the 12-month retention rate was 55% (29). This is likely due to the refractory nature of epilepsy in these European studies, as PWE had failed on a median of 6 and 4 ASMs, respectively, before starting PER treatment (28, 29).

Our findings are consistent with previous works suggesting that the response to ASMs is greater in the early than in the late add-on setting (30, 31) and with previous studies of the ASMs brivaracetam and lacosamide (32, 33). In PWE treated with brivaracetam for 12 months as early (one or two previous ASMs) or late (three or more previous ASMs) add-on therapy, ≥50% responder rates were 60.3 and 34.3% in the early and the late add-on subgroups, respectively (*p* < 0.001), and seizure freedom rates were 31.7 and 10.9%, respectively (*p* < 0.001) (32). Similarly, in PWE treated with lacosamide for 12 months as early (add-on to first ASM monotherapy) or late (add-on to one to three concomitant ASMs after at least two previous ASMs) adjunctive therapy, ≥50% responder rates were 70.3 and 50.4% in the early and late add-on subgroups, respectively, and seizure freedom rates were 37.5 and 14.9%, respectively (33). Both studies concluded that the two ASMs were efficacious in both subgroups, supporting the use of the ASMs as early add-on therapy.

The significant differences in treatment outcomes between the two subgroups are likely to reflect the different stage and/or severity of epilepsy in the early and late add-on subgroups, as indicated by the notable differences in demographic and epilepsy-related baseline characteristics between the subgroups. Since the subgroups were defined in terms of the number of ASMs with which PWE had previously failed to achieve adequate seizure control, it is unsurprising that those in the early add-on subgroup had characteristics associated with an earlier stage of disease than those in the late add-on subgroup, such as shorter duration of epilepsy, lower monthly seizure frequency and lower number of concomitant ASMs. The significantly lower proportions of PWE with intellectual disability or psychiatric comorbidity in the early vs. late add-on subgroup may likewise reflect an earlier disease stage in the former subgroup, since both types of comorbidity are impacted by disease- and treatment-related factors that are likely to increase as the disease progresses (34, 35). It is also possible that, as the age at epilepsy onset was significantly lower in late vs. early add-on subgroup, the late add-on subgroup included a higher proportion of PWE who had childhood syndromes associated with intellectual disability. However, there were other differences that were also notable. As previously mentioned, PWE in the early vs. late add-on subgroup had a significantly later onset of epilepsy, indicating that a higher proportion of elderly patients were included in the early add-on group epilepsy and there is evidence that elderly PWE have a better prognosis as they respond better to ASM treatment (36–38). The etiology of the subgroups also differed significantly, the early add-on subgroup containing a higher proportion of PWE with a genetic etiology. This may reflect the fact that the PERMIT cohort contained a relatively high proportion of individuals with IGE, since idiopathic epilepsies may respond more favorably to ASM therapy than other epilepsy etiologies (25, 39). The significantly higher proportion of males to females in the early vs. late add-on subgroup is also noteworthy, although reasons for this are unclear. One possible explanation is the higher percentage of PWE with IGE (genetic etiology) in the early vs. late add-on subgroup, as women of childbearing age are likely to avoid using valproate (the proportion of PWE previously treated with valproate was 23.6% in the early add-on subgroup vs. 64.8% in the late add-on subgroup). The findings from this study are consistent with multivariable regression analyses conducted for the overall PERMIT study population, which demonstrated that the baseline factors most associated with response to PER treatment and seizure freedom included lower number of previous ASMs; also, lower number of concomitant ASMs at baseline, higher age at onset of epilepsy, the presence of a genetic etiology, and the absence of psychiatric comorbidity (25).

In the current study there were also marked differences between the two subgroups in terms of treatment patterns. Most notably, PER dosing was significantly lower at the last visit in the early vs. late add-on subgroup, mirroring the significantly greater effectiveness observed in the early vs. late add-on subgroup, and consistent with PWE in the early add-on group being less refractory to treatment than those in the late add-on subgroup. The significant decrease in the number of concomitant ASMs used in both subgroups during the study does, however, indicate that PER was effective regardless of disease stage. It is also important to consider that the lower PER dosing used in the early add-on subgroup may have contributed to the superior safety/tolerability observed in the early vs. late add-on subgroup.

Several studies investigating PER as an early or first add-on therapy in clinical practice were included in the PERMIT study (40–48). However, since the PERMIT study was conducted, several other studies of PER as first add-on therapy have been conducted, in both the clinical trial and clinical practice settings. In the Phase 4, prospective, open-label, single-arm cohort Fycompa^®^ as first Add-on to Monotherapy in patients with Epilepsy (FAME) trial, PER was added to monotherapy in 85 PWE with focal-onset seizures, with or without focal to bilateral tonic-clonic seizures (FBTCS) (49). The ≥50% responder rate and seizure freedom rate during the 24-week maintenance period were 80.0 and 47.1%, respectively (49). In the current study, the 6-month responder and seizure freedom rates for focal seizures in the early add-on subgroup were 61.4 and 28.0%, respectively, while the corresponding rates for total seizures in the *first* add-on subgroup were 71.6 and 42.2%, respectively. As in the current study, the most frequently reported treatment-emergent AEs in FAME were dizziness and somnolence (also headache) (49). Responder and seizure freedom rates were sustained during the 3-year FAME extension study (50). COM-PER was a Phase 4, retrospective, multicentre, observational study that compared the 12-month retention, effectiveness and tolerability of PER when used as a first add-on treatment (*n* = 21) or late add-on treatment (defined as failure of >3 previous ASMs; *n* = 60) in adult PWE with focal-onset seizures, with or without FBTCS (51). As in the current study, the 12-month retention rate was significantly higher for the first vs. late add-on subgroup (90.5 vs. 48.3%; *p* = 0.001), as were the 12-month ≥50% responder rate (85.7 vs. 28.3%; *p* < 0.001) and seizure freedom rate (71.4 vs. 13.3%; *p* < 0.001) (51). Unlike in the current study, tolerability did not differ significantly between the first vs. late add-on cohorts (51). The most commonly reported AEs (≥5% of PWE in either subgroup) were dizziness, irritability and somnolence (51). In both FAME and COM-PER, PER was shown to be particularly effective in PWE with FBTCS (49, 51). In the clinical practice setting, a retrospective, observational, multicentre, longitudinal study assessed the effectiveness and tolerability of PER at 3, 6 and 12 months when used as the only add-on to ASM monotherapy in PWE with focal and generalized epilepsy aged >12 years (52) and a subanalysis was conducted to compare the use of PER as an early single add-on (after 0 or 1 previous add-on therapies) or late single add-on (after ≥2 previous add-on therapies) (52). Retention, responder and seizure freedom rates were similar between subgroups with the exception of the ≥50% responder rate (66 vs. 53%; *p* = 0.05) and the seizure freedom rate (42 vs. 25%; *p* = 0.005) at 3 months, both significantly higher in the early vs. late add-on subgroup (52). The most frequently reported AEs were dizziness/vertigo and behavioral changes at all timepoints (52). Finally, a single-center, open-label study compared the efficacy of PER in individuals with a diagnosis of mesial temporal lobe epilepsy when used as first add-on therapy (due to inefficacy of a first ASM; *n* = 20) vs. late add-on therapy (due to inefficacy of ≥2 ASMs; *n* = 17) (53). PER efficacy was assessed at 3 and 12 months (53). Consistent with the current study, the proportion of PWE achieving either >50% seizure frequency reduction or seizure freedom was significantly higher in the first vs. late add-on subgroup after 3 months (70.0 vs. 23.5%; *p* = 0.005) and 12 months (70.0 vs. 29.4%; *p* = 0.014) (53). Taken together, the findings of these studies are broadly consistent with those of the current PERMIT analysis, in terms of effectiveness and tolerability, demonstrating that although PER is an effective add-on therapy regardless of when it used, it is more effective when used as an early add-on therapy than a late add-on therapy. Furthermore, they are consistent in demonstrating that add-on PER is effective in treating both focal and generalized seizures (particularly the latter), consistent with PER being a broad-spectrum ASM.

As a *post-hoc* analysis of PERMIT, this study has acknowledged limitations. PERMIT was a retrospective pooled analysis of a heterogeneous group of studies that differed in terms of objectives and information reported, and therefore did not have complete data available for all PWE at all timepoints (25). Since most studies included in PERMIT were uncontrolled retrospective analyses of cases, they may have suffered from selection bias against PWE judged to be at risk of experiencing known side effects of PER treatment (25). Pooled analyses of real-world studies may also over-estimate the clinical value of the agent under investigation, since it is not possible to assess to potential impacts of factors such as participant selection, regression to the mean, and the bias caused by the early discontinuation of subjects who fail to respond to the intervention (54). Moreover, since there was no control group in PERMIT, it was not possible to assess the effects of PER vs. placebo/no drug or the passage of time (54). Observation timepoints of some studies included in PERMIT did not match those used for evaluation (i.e., 3, 6 and 12 months), which may have affected the findings for some assessments. It is also important to note that individual subject data were not reviewed systematically in the current study (although reviewed by the investigators of the original studies included in PERMIT), and that seizure freedom was defined as no seizures since at least the prior visit (rather than no seizures since initiation of PER treatment), which could have been 3 or 6 months, depending on the timepoint concerned. An additional limitation is that the baseline and disease characteristics of the two subgroups indicate that disease severity was greater in the late vs. the early add-on subgroup. Finally, it should be considered that PWE in the late add-on subgroup were receiving significantly more concomitant ASMs than those in the early add-on subgroup, which might have contributed to the higher rate of AEs in the former subgroup (55).

In summary, the current study demonstrated that PER was effective and generally well-tolerated when used to treat PWE with focal seizures and GTCS in clinical practice, regardless of whether it was initiated as early or late add-on therapy, but it was significantly more effective and better tolerated when initiated as an early vs. late add-on therapy. Overall, the findings support the use of PER as a broad-spectrum, early add-on therapy for use in PWE with focal and generalized seizures. As pharmacological treatment should be started as soon as possible to reduce seizure recurrence, achieve seizure control and improve PWE's quality of life, selecting a broad-spectrum ASM such as PER as early add-on treatment might ensure better treatment outcomes.

## Data availability statement

The raw data supporting the conclusions of this article will be made available by the authors, upon reasonable request.

## Ethics statement

Ethical review and approval was not required for the study on human participants in accordance with the local legislation and institutional requirements. Written informed consent from the patients/participants or patients/participants' legal guardian/next of kin was not required to participate in this study in accordance with the national legislation and the institutional requirements.

## Author contributions

CL, ES, AS, JJR-U, RS, XR-O, SA, PB, ET, RM, RS-F, and VV: contributed equally to the drafting and critical review of the manuscript. VV: responsible for data extraction and analysis with a statistician's support. All authors contributed to the article and approved the submitted version.
