# Supplementary material for: Perampanel outcomes at different stages of treatment in people with focal and generalized epilepsy treated in clinical practice: Evidence from the PERMIT study
Source: Front Neurol. 2023 Mar 30;14:1120150. doi: 10.3389/fneur.2023.1120150 (PMC10098362; doi:10.3389/fneur.2023.1120150)

**Supplementary material**

**Supplementary Figure S1. Proportions of PWE with worsening seizure frequency at Month 3, Month 6, Month 12 and the last visit in the early and late add-on subgroups for (A) Total seizures, (B) Focal seizures and (C) GTCS (Effectiveness population).** GTCS, generalised tonic-clonic seizures


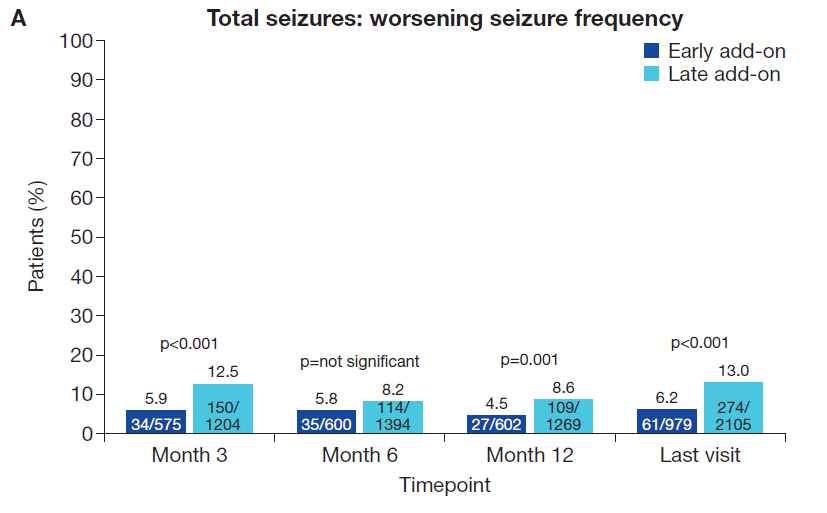


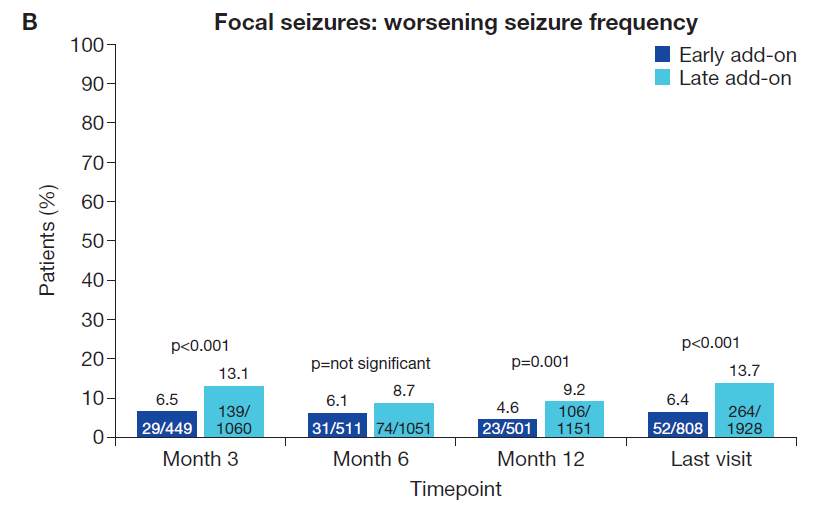


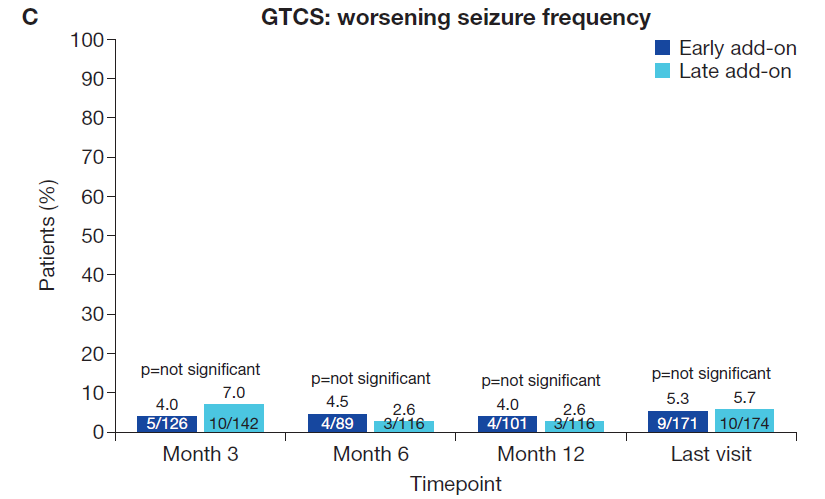


**Supplementary Figure S2. Retention and effectiveness in the first and second add-on subgroups: (A) Retention rate (Retention Population) (B) Responder rate (total seizures; Effectiveness Population) and (C) Seizure freedom rate (total seizures; Effectiveness population).** Response was defined as ≥50% seizure frequency reduction from baseline. Seizure freedom was defined as no seizures since at least the prior visit. CI, confidence interval


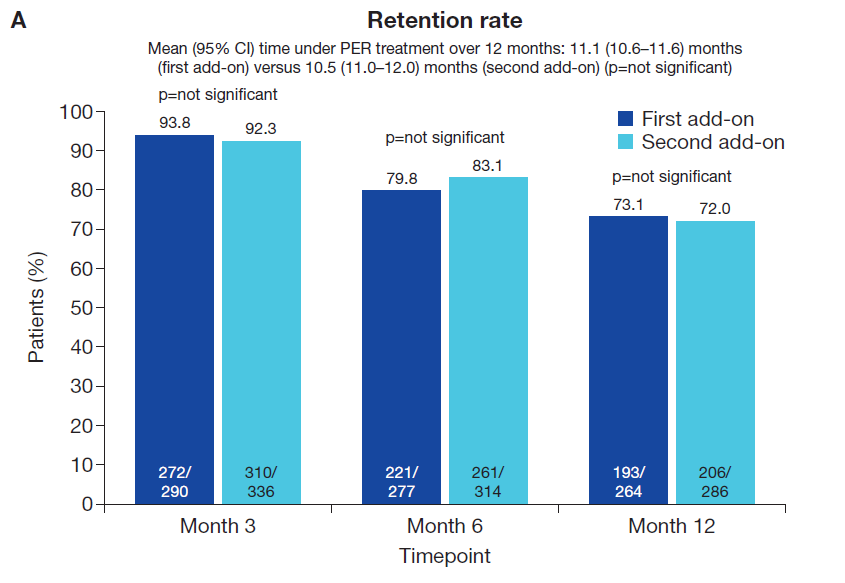


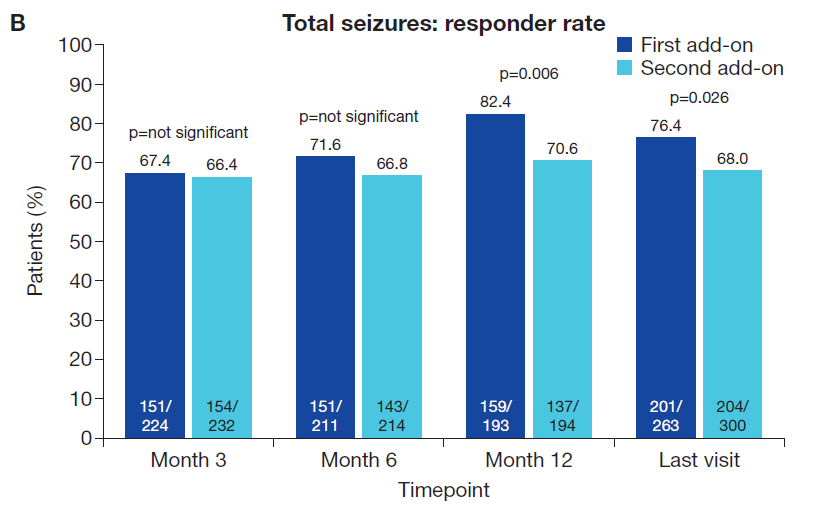


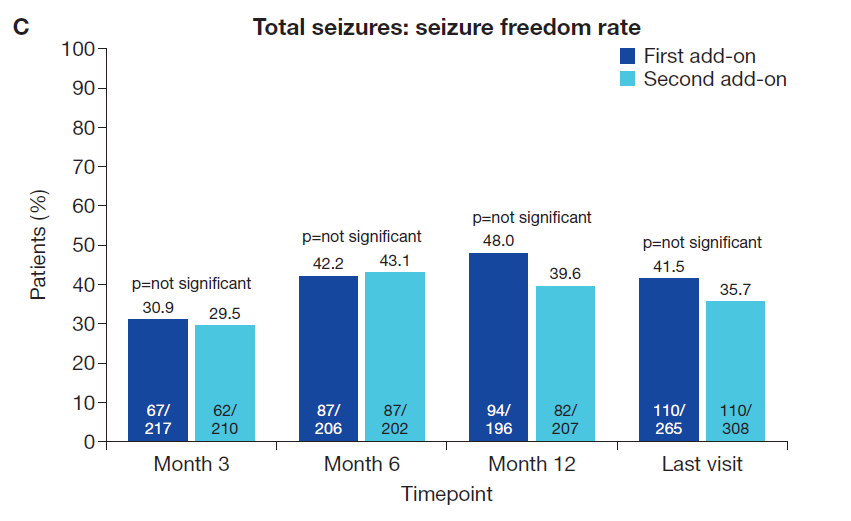

Supplement: Supplementary file 1 [file Table_1.DOCX]
